# Early results of urethral dose reduction and small safety margin in intensity-modulated radiation therapy (IMRT) for localized prostate cancer using a real-time tumor-tracking radiotherapy (RTRT) system

**DOI:** 10.1186/1748-717X-9-118

**Published:** 2014-05-21

**Authors:** Shinichi Shimizu, Kentaro Nishioka, Ryusuke Suzuki, Nobuo Shinohara, Satoru Maruyama, Takashige Abe, Rumiko Kinoshita, Norio Katoh, Rikiya Onimaru, Hiroki Shirato

**Affiliations:** 1 Department of Radiation Medicine, Hokkaido University Graduate School of Medicine, Sapporo, Japan; 2 Department of Radiation Oncology, Hokkaido University Hospital, Sapporo, Japan; 3 Department of Renal and Genitourinary Surgery, Hokkaido University Graduate School of Medicine, Sapporo, Japan; 4 Department of Urology, Hokkaido University Hospital, Sapporo, Japan

**Keywords:** Prostate cancer, Radiotherapy, Intensity-modulated radiation therapy, Image-guided radiation therapy, Adverse event

## Abstract

**Background:**

We prospectively assessed the utility of intensity-modulated radiation therapy (IMRT) with urethral dose reduction and a small margin between the clinical target volume (CTV) and the planning target volume (PTV) for patients with localized prostate cancer.

**Methods:**

The study population was 110 patients in low- (14.5%), intermediate- (41.8%), and high-risk (43.6%) categories. Three gold fiducial markers were inserted into the prostate. A soft guide-wire was used to identify the urethra when computed tomography (CT) scan for treatment planning was performed. A dose constraint of V70 < 10% was applied to the urethral region. Margins between the CTV-PTV were set at 3 mm in all directions. Patients were treated with 70 Gy IMRT in 30 fractions (D95 of PTV) over 7.5 weeks. The patient couch was adjusted to keep the gold markers within 2.0 mm from their planned positions with the use of frequent on-line verification.

**Results:**

The median follow-up period was 31.3 (3.2 to 82.1) months. The biochemical relapse-free survival (bRFS) rates at 3 years were 100%, 93.8% and 89.5% for the low-, intermediate-, and high-risk patients, respectively. The incidences of acute adverse events (AEs) were 45.5% and 0.9% for grades 1 and 2, respectively. The late AEs were grade 1 cystitis in 10.0% of the patients, rectal bleeding in 7.3%, and urinary urgency in 6.4%. Only three patients (2.7%) developed grade 2 late AEs.

**Conclusions:**

On-line image guidance with precise correction of the table position during radiotherapy achieved one of the lowest AEs rates with a bRFS equal to the highest in the literature.

## Background

The use of image-guided radiation therapy (IGRT) equipment has been shown to reduce the adverse events on organs at risk (OAR) near the clinical target volume (CTV) in intensity-modulated radiation therapy (IMRT) [1,2]. For prostate cancer, it has been demonstrated that the rate of complications involving the rectal wall (an OAR near the CTV) can be reduced by using IGRT equipment [3]. However, it has been difficult to reduce the dose to the OAR within the CTV with IMRT because of the uncertainty in localization [4]. Complications caused by the urethra in the prostate, which is regarded as an OAR within the CTV, may be avoided by reducing the dose to the urethra with precise real-time IGRT and by correcting the table position during radiotherapy to reduce intra-fractional positional errors.

We have reported a real-time tumor-tracking radiation therapy (RTRT) system [5] which uses the implantation of fiducial markers and two sets of room-mounted fluoroscopy. This system can maintain accuracy within nearly 2 mm during radiotherapy by reducing the intra-fractional as well as inter-fractional errors, and it could also be called on-line image-guided radiotherapy with intra-fractional correction (intra-IGRT) [6]. We have not yet encountered any situation in which the precision of intra-IGRT would be required rather than non-intra-IGRT technologies that do not improve intra-fractional accuracy but do improve inter-fractional accuracy (inter-IGRT).

We postulated that a combination of intra-IGRT and IMRT would enable a reduction of the dose to the urethra in the prostate while maintaining the required dose to the prostate, compared to inter-IGRT technologies which only have inter-fractional image guidance and table correction capability.

To test our hypothesis, we started a prospective study in 2004. Newly devised procedures in treatment planning were developed and implemented in the treatment protocol to identify soft structures (e.g., bladder neck and urethral tract) in the prostate which are movable during radiotherapy. The primary end-point was the incidence of AEs. A secondary end-point was biological relapse-free survival (bRFS).

## Methods and materials

### Patients

One hundred-ten consecutive localized prostate cancer patients (median age, 70 years; range 52 to 79) treated between Dec. 2004 and Nov. 2011 were included. The characteristics of the patients are shown in Table 1. They were categorized into low- (14.5%; n = 16), intermediate- (41.8%; n = 46), and high-risk (43.6%; n = 48) groups according to the National Comprehensive Cancer Network (NCCN) guidelines [7]. All but three patients received the total dose as planned in the protocol; one received 29 fractions, and two received 28 fractions instead of 30 fractions because of the comorbidity of severe vascular diseases. The median follow-up period was 31.3 months (3.2 to 82.1) for all patients. Following local Ethics Committee (the ethical committee of Hokkaido University Graduate School of Medicine) approvals patients were enrolled this study and written informed consent was obtained from all the patients prior to treatment.

**Table 1:** Characteristics of the 110 localized prostate cancer patients

**Characteristic**		**No. of patients (%)**
Age		52–79 (median 70.0)
Risk group	Low	16 (14.5%)
	Intermediate	46 (41.8%)
	High	48 (43.6%)
T-Stage (UICC 6th)	T1c-T2a	81
	T2b	10
	T3a-T3b	19
Initial PSA value	<10 ng/mL	60 (54.5%)
	10–19.9 ng/mL	26 (23.6%)
	≥20 ng/mL	24 (21.8%)
Gleason score	5–6	33 (30.0%)
	7	41 (37.3%)
	8–10	36 (32.7%)
History of hormonal therapy	(−)	76 (69.1%)
	(+)	34 (30.9%)
Dose/fraction	70 Gy/30 Fr(D95)	101
	75 Gy/30 Fr(Iso)	5
	65.3 Gy/28 Fr(D95)	3
	67.5 Gy/29 Fr(Iso)	1

### Treatment

**Figure 1:**
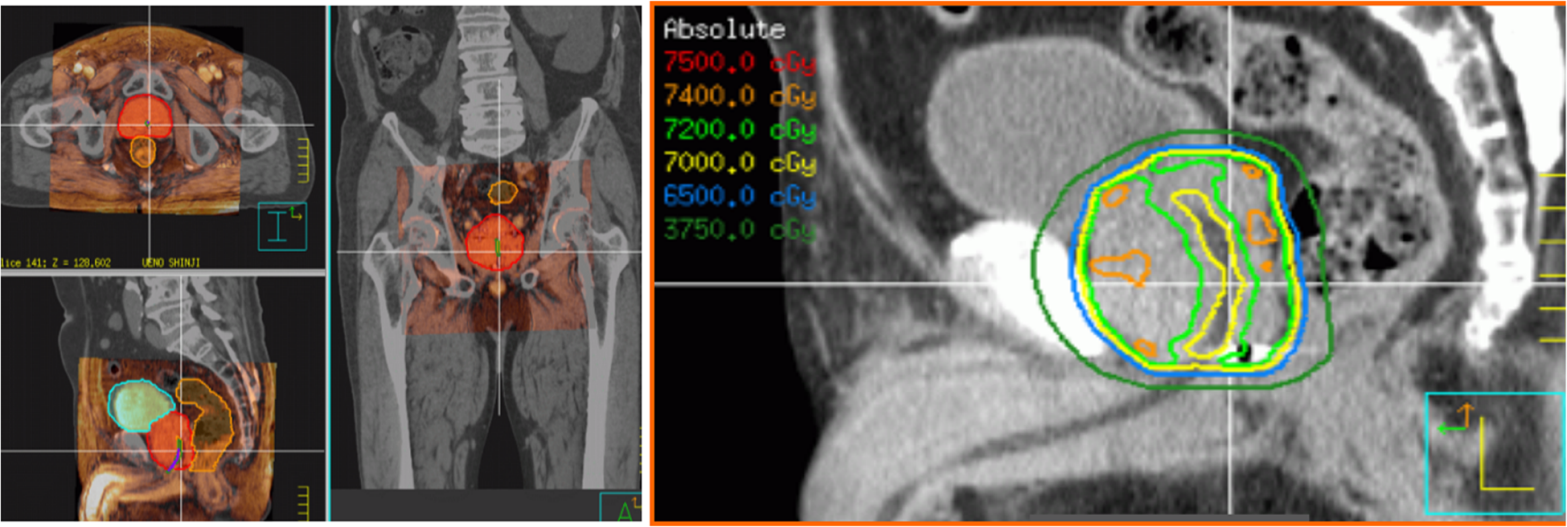
**MRI image fusion for treatment planning and dose distribution for urethra dose controlled IMRT planning.** MRI image fusion is used for treatment planning (left image). IMRT planning is performed with the constraint that urethra dose will not exceed V70Gy > 10% generally (right image).

Three gold fiducial markers were inserted into the prostate of each patient, for tumor localization [8]. The three markers (2.0 mm dia.) were inserted into the patient's prostate gland one week before the CT for treatment planning. To ensure a constant bladder volume, an intravesical instillation of 100 mL of sterile normal saline was followed by CT scanning of the small pelvis with the patient in the supine position on a flat carbon table. A soft guide-wire (0.46 mm dia.) was inserted into the urethra through a urethral catheter (4.0 mm dia.) when the CT scan was performed. Magnetic resonance imaging (MRI) was also obtained with the urethral catheter in place (Figure 1 left).

In total, (a) CT images *with the catheter*/*guide*-*wire* in the urethra tract, (b) CT images *with the guide*-*wire only* in the urethra tract, and (c) MRI *without the catheter*/*guide*-*wire* were all used for each patient's treatment planning. All of the images were transferred to a treatment planning system (TPS), XiO (CMS, St. Louis, MO from 2004.4 to 2007.9) or Pinnacle^3^ (Hitachi Medical Co., Tokyo from 2007.10 to 2011.11) and fused according to the location of the three implanted markers. The image fusion was performed according to the fiducial markers, not by the bony structures, nor by the soft tissues. For the MR images, spotty signal voids caused by the gold markers were used to match them to the high-density signals on CT images. The gold markers did not make any apparent distortion of MRI.

The contours of the prostate gland including the urethra were defined as the CTV for all patients. The CTV included the prostate and seminal vesicles for the high-risk group and for intermediate-risk patients who had any two of the following three factors: T2b or higher T stage, moderately differentiated adenocarcinoma in pathological diagnosis, and initial prostate-specific antigen (PSA) ≥10. The planning target volume (PTV) was determined by a 3D expansion of the CTV with the addition of a 3-mm margin.

The total dose of radiation treatment was prescribed at the dose covering 95% of the PTV (D95). Seventy Gy 7-field IMRT in 30 fractions (D95 of PTV) over 7.5 weeks was used for almost all the patients. This dose was selected to be equal to our previous treatment, 75 Gy at 2.5 Gy per fraction prescribed at the isocenter. Six patients were prescribed the dose at the isocenter at the beginning of this treatment. For four patients, the fraction number was reduced one or two times to satisfy the dose constraint. The urethra as an OAR was contoured in 2 mm diameter Region of interest (ROI) with reference to the guide-wire that appeared on the CT images. A margin of 2 mm was applied to the urethra (OAR) to determine the planning organ at risk volume (PRV) (OAR + margin). A dose constraint of V70Gy < 10% was applied to the PRV of the urethral region (4 mm dia.) along the catheter in the prostate represented on the CT images (Figure 1 right). The dose constraint was *not* used for the location when a discrepancy between (a) *the catheter*/*guide*-*wire* in CT and (b) *the guide*-*wire only* in CT was observed; this is because a discrepancy suggests a soft and movable nature of the location and a higher risk of misalignment of the urethra during irradiation. Bladder neck was the most movable location; details of the anatomical changes observed will be presented elsewhere. Other dose constraints were rectal V60 Gy < 20%, rectal V37.5 Gy < 50%, and bladder V37.5 Gy < 30%.

The detail of the on-board imaging used in this study has been described previously [8]. Basically observation through the diagnostic X-ray can be made real time during therapeutic beam delivery. And calculations for the localization of the target accuracy were made 7.6 times on average at one-day treatment. The incidence of the couch adjustment required were differ patients to patients; 6 to 68 (median 19) times position adjustment during the 30 fractions treatment for a patient.

All radiotherapy was performed *without the catheter*/*guide*-*wire* insertion. The catheter/guide-wire was not inserted during radiotherapy since the guide-wire was shown to represent the urethral position only in our preparatory studies during image acquisition. All seven ports were treated in each daily treatment. Using frequent on-line verification with the RTRT system during treatment at least at the start of every portal irradiation, the patient couch was adjusted on-line to keep the gold markers within 2.0 mm from their planned positions. For the patients who moved during irradiation, continuous fluoroscopic observation was used and the irradiation was automatically interrupted when the marker moved more than 2.0 mm. Patients are asked to void a few hours before every radiotherapy. The details of this procedure were as reported [6,8].

Hormonal treatment was accepted as a neo-adjuvant and/or concomitant treatment during radiotherapy for 34 patients (27 high-risk, 6 intermediate-risk, and 1 low-risk).

### Assessments and statistical analysis

Patients were followed by both the referring urologist and a radiation oncologist every 3 months after the treatment. After 5 years, the follow-up interval was changed to once a year. Acute and late AEs were scored according to the Common Toxicity Criteria Adverse Events version 4 (CTCAE v4.0) scale. Acute AE was defined as an AE originating within 90 days from the completion of radiation therapy. Late AE was defined as AE appearing more than 90 days from the completion of radiation therapy. Biochemical relapse-free survival (bRFS) was defined as the time between the first day of radiotherapy and the date of biochemical failure as defined by the Phoenix definition (nadir of PSA + 2 ng/mL) or death from any cause.

A comparative study of the doses to the urethra and CTV was added as an additional study for 77 patients whose treatment plans were made with the Pinnacle^3^ TPS after 2007. Each minimum, maximum, and mean dose was compared with the adverse events. All statistical analyses were performed using JMP 9.0.3 (SAS Institute, Cary, NC, USA).

## Results

The incidences of acute gastrointestinal (GI) AEs were 7 (6.4%) for grade 1 (Table 2). A late grade 1 GI AE was observed in 8 (7.3%) patients. No acute and late grade 2 or higher-grade GI AE was observed.

**Table 2:** **Rates of acute and late adverse events among prostate cancer patients** (**n** = **110**)

**Grade**		**Acute**				**Late**				
		**0**	**1**	**2**	**3**	**0**	**1**	**2**	**3**	
GI	Total	103	7	0	0	102	8	0	0	
		93.6%	6.4%	0%	0%	92.7%	7.3%	0%	0%	
	Rectal mucositis/pain			7						
	Rectal bleeding							8		
GU	Total	55	44	1	0	90	17	3	0	
		50.0%	40.0%	0.9%	0%	81.8%	15.5%	2.7%	0%	
	Frequency			30	1					
	Cystitis noninfective			27				11	1	
	Retention/obstruction			12					1	
	Urgency			14	1			7	1	
	Urethral tract pain			12						

GI, gastrointestinal; GU, genitourinary. Acute (AE): originating within 90 days from the completion of radiation therapy. Late (AE): appearing more than 90 days from the completion of radiation therapy.

The incidences of acute genitourinary (GU) AEs were 44 (40.0%) for grade 1 and 1(0.9%) for grade 2. Acute grade 1 voiding frequency and cystitis were observed in 30 (27.3%) and 27 (24.5%) patients, respectively. Acute grade 2 voiding frequency and urgency were observed in 1 (0.9%) and 1 (0.9%) patient, respectively. Late grade 1 and grade 2 GU AEs were observed in 17 (15.5%) and 3 (2.7%) patients, respectively. No grade 3 or higher-grade acute or late toxicity was observed.

**Figure 2:**
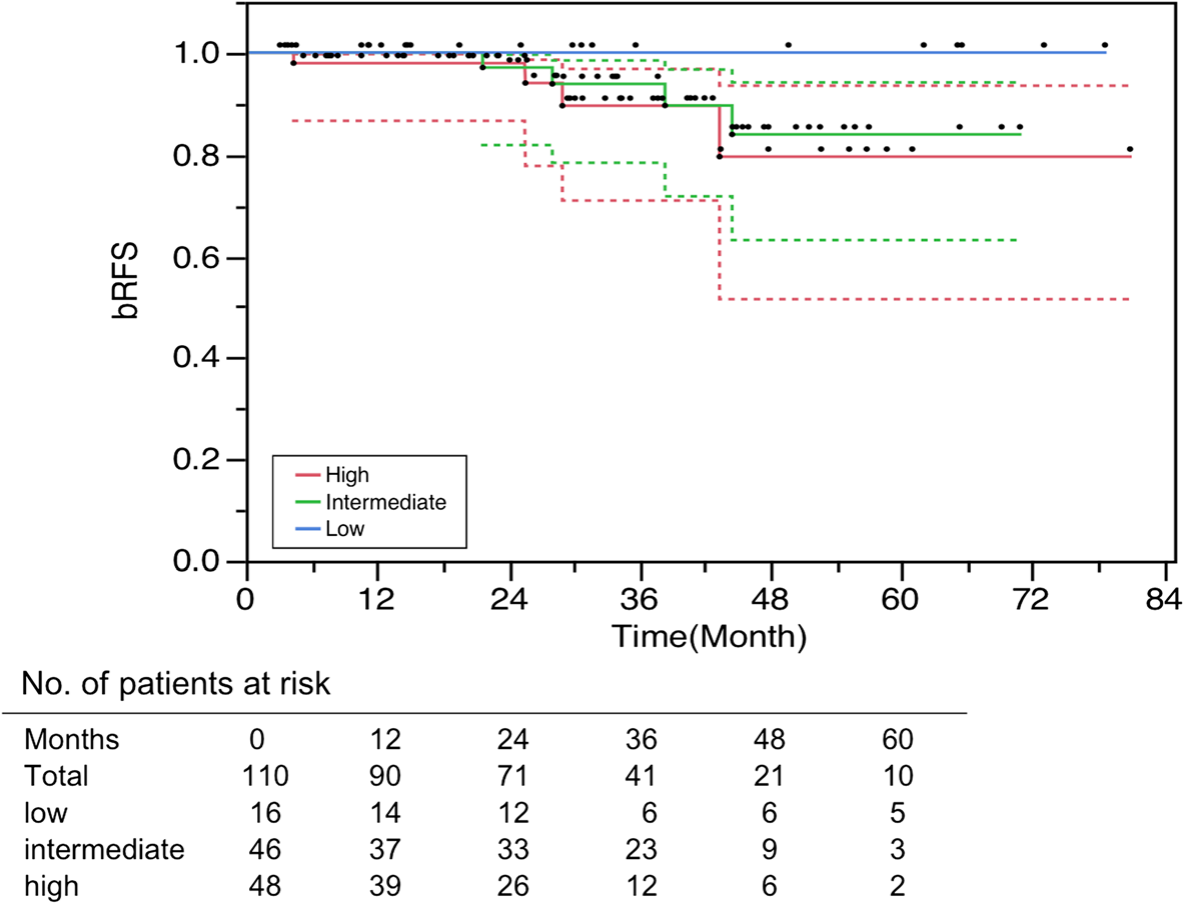
**Overall bRFS (biological relapse-free survival) for all 110 prostate cancer patients.**

The bRFS rates at 3 years were 100%, 93.8% and 89.5%; at 5 years were 100%, 84.0% and 79.6% for the low-, intermediate-, and high-risk patients respectively (Figure 2). Hormonal treatment was accepted as a neo-adjuvant and/or concomitant treatment during radiotherapy for 34 patients (27 high-risk, 6 intermediate-risk, and 1 low-risk).

**Figure 3:**
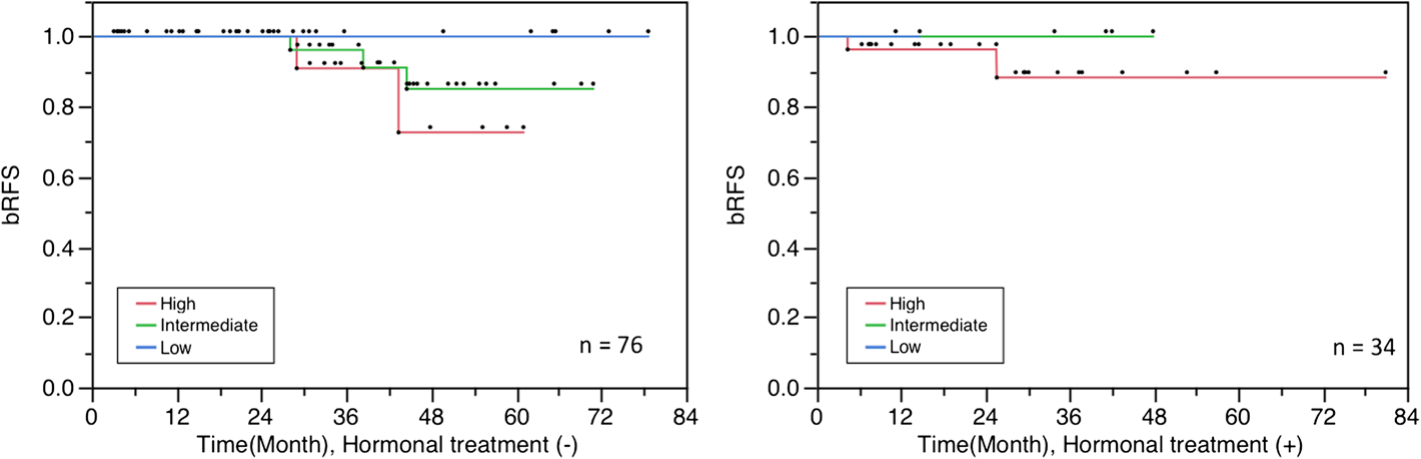
**Overall bRFS for patients with**/**without hormonal therapy.**

Of the 110 patients, 2 patients died; 1 patient from distant metastasis on day 331 and another due to renal cancer with metastasis to the lung and bone on day 441 without biochemical failure (Figure 3).

**Figure 4:**
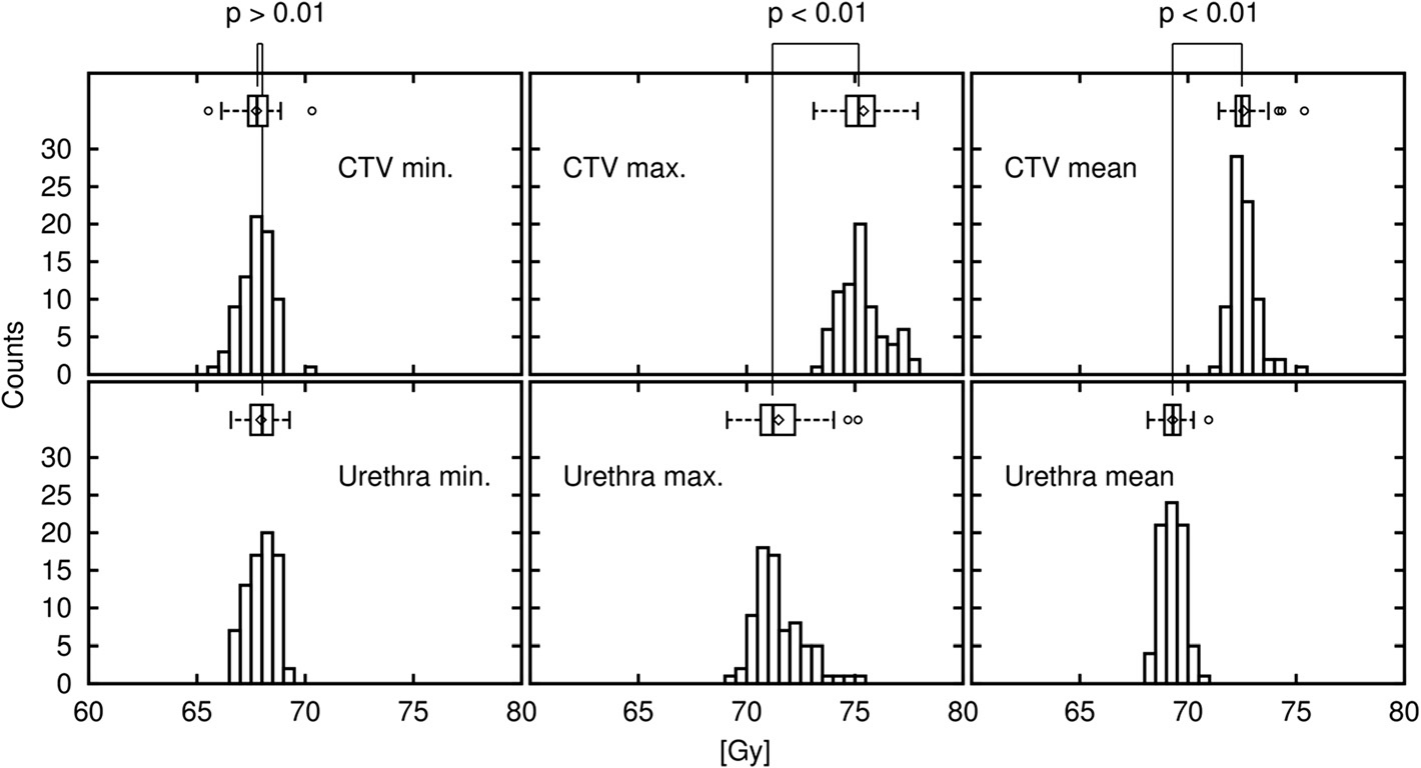
Histogram of the CTV dose and the urethra dose.

In the comparison of the dose to the CTV and the urethra, there was no significant difference between the minimum dose of CTV (67.76 Gy ± 0.75 Gy) and that of urethra (67.96 ± 0.65 Gy) (p = .073). There was significant difference between the maximum dose of CTV (75.40 ± 1.23 Gy) and that of urethra (71.47 ± 1.16 Gy) (p < 0.001) and the mean dose of CTV (72.58 ± .65 Gy) and urethra (69.29 ± 0.53 Gy) (p < 0.001), respectively. The medical mean dose to the PTV including the urethral region ranged from 71.34 to 72.52 Gy (Figure 4). There was no relationship between the minimum or mean dose and the bRFS, or between the maximum dose of the CTV or urethral region and the urethral adverse events in the dose range in our study.

## Discussion

Kupelian et al. published excellent results of their hypofractionated IMRT study in which the nominal prescription dose was 70 Gy in 28 fractions [9]. In a linear-quadratic model, the α/β ratio for prostate cancer was reported to be smaller than those of acute-responding tissues and estimated as 1.5 by Brenner et al. [10]. If we assume that the α/β ratio is 1.5 for prostate cancer, 70 Gy in 28 fractions is equivalent to a biological effective dose (BED) of 187 Gy, or 85 Gy using a daily dose of 1.8 Gy. Our schedule is similar to their schedule and to their biological dose, since the mean dose of CTV in the present study, 72.58 Gy in 30 fractions, is equivalent to a BED of 190 Gy or 86.4 Gy in 48 fractions.

The primary end-point of our study was the incidence of adverse events. Kupelian et al. showed that the acute GI toxicity was grade 1 in 40% of their patients and grade 2 in 9%; late GI toxicity was grade 1 in 5.9%, grade 2 in 3.1%, grade 3 in 1.3%, and grade 4 in 0.1%. We showed that acute GI toxicity was grade 1 in 6.4% of our patients, and late GI toxicity was grade 1 in 7.3%; no grade 2 or higher toxicity was observed. Kupelian et al. reported that acute GU toxicity was grade 1 in 48% of their patients, grade 2 in 18%, and grade 3 in 1%; late GU toxicity was grade 1 in 4.3%, grade 2 in 5.1%, and grade 3 in 0.1%. In our patient population, we observed that the acute GU toxicity was grade 1 in 40% and grade 2 in 0.9%, and late GU toxicity was grade 1 in 15.5% and grade 2 in 2.7%. No grade 3 or higher toxicity was observed.

Table 3 illustrates the results of hypofractionated radiotherapy for prostate cancer in recent publications. The volumes of CTV, which depend on the treatment policy, and the lengths of PTV margin, which depend on the positioning accuracy, were also shown in the table. At a glance, the incidence of complications seems to be related to the volume of CTV and the length of PTV margin [9,11-13]. As for the toxicity scale, Fonteyne et al. mentioned that there is a need for a uniform toxicity scoring system for our radiotherapy community, because each toxicity scale such as RTOG, SOMA/LENT and CTC toxicity scale lacks important symptoms [14]. They also described that the omission of the symptoms leads to underreporting of toxicity and the incidence of late GU toxicity is influenced by the toxicity scale that is used. So it is difficult to compare our results with those of other institutions, the very low incidence of toxicity in the present study might be attributable to the small PTV-CTV margin, the reduction in the urethral dose, and the reduction in intra-fractional error due to organ motion. Further observation for more long period and more in number, however, is required for obtaining firm conclusion.

**Table 3:** Overview of hypofractionated radiotherapy for prostate cancer

**Author(s) (year) [Referense no.]**		**Arcangeli G (2011) **[11]	**Fonteyne et al. (2012) **[12]	**Kupelian et al. (2007) **[9]	**Dearnaley et al. (2012) **[13]		**Corrent study (2014)**
Nominal dose (Gy)/no. of fractions		62/20	56/16	70/28	60/20	57/19	70/30
Number of patients		83	113	770	153	151	110
Follow-up period (months)		35	47	45	51		31
CTV for T1-2 & low risk		Prostate & seminal vesicles	Prostate only	Prostate only	Prostate + base of seminal vesicles		Prostate only
CTV for T3 & high risks			Prostate & seminal vesicles	Prostate & seminal vesicles	Prostate & seminal vesicles		Prostate & seminal vesicles
PTV margin (mm)	Craniocaudal	10	3-10	5	5	5	3
	Anterior	10		5	5	5	3
	Lateral	10		8	5	5	3
	Posterior	6		4	0	0	3
Early GU (%)	G2	46	38	18	8	7	1
	G3	1	0	1	0	0	0
	G4	0		0	0	0	0
Early GI (%)	G2	35	10	9	2	1	0
	G3	0	4	0	0	0	0
	G4	0		0	0	0	0
Late GU (%)	G2	8	10	5	2	0	3
	G3	0	4	1	0	0	0
	G4	1		0	0	0	0
Late GI (%)	G2	14	7	3	4	1	0
	G3	1	2	1	0	0	0
	G4	0		0	0	0	0
Biochemical relapse free survival 5 yrs (3 yrs) %	Low	-	98	94	-	-	100 (100)
	Intermediate	-	93	83	-	-	84 (94)
	High	-	82	72	-	-	80 (90)

Our secondary end-point was the bRFS. The 5-year rates for the patients with low-, intermediate-, and high-risk disease were 97%, 93%, and 75%, respectively in Kupelian's study [9]. Although the confidence interval is still too large, the present bRFS results for the low-, intermediate-, and high-risk disease risk groups were encouraging to be as effective as Kupelian et al.'s outcome and those in other reports so far [2-4,11,15,16]. Kupelian et al. used an ultrasound system to reduce the inter-fractional set-up error and adapted a PTV-CTV margin of 4 mm posteriorly, 8 mm laterally, and 5 mm in all other directions. Crehange et al. recently showed that a 5-mm margin in all directions is also acceptable with the use of an ultrasound system [3].

To the best of our knowledge, the present study is the first one suggesting that image-guided IMRT with an only 3-mm PTV-CTV margin may achieve a bRFS rate equivalent to those of previous series using the same end-point. In this study, we restricted the urethral dose by using V70Gy < 10% as the dose constraint for its PRV, to reduce hot spots in the urethral region. It should be noted that we kept the minimum CTV dose at 67.76 Gy ± 0.75 Gy (which is equivalent to 85 Gy using 1.8 Gy), and we did not reduce the urethral dose further, to avoid increasing the risk of tumor relapse around the urethra.

Zelefsky et al. reported reductions in acute and late GU toxicity without compromising PSA relapse-free survival achieved by the implantation of fiducial markers and inter-IGRT [2]. They attributed the reduced incidence of urinary toxicity to the reduction of the dose and volume to the bladder and bladder neck region. Our results are consistent with their suggestion that the reduction of doses to these regions is important to reduce frequent urination and urgency. Obstruction of voiding is also known as a late GU toxicity [10]. Coen et al. suggested the possibility of reducing the dose to the urethra as a new strategy to improve radiotherapy for prostate cancer [17]. The findings of the present study suggest that a treatment technique that reduces the dose to the urethra running through the prostate gland can reduce the late toxicity of urethral stricture and obstruction.

Vainshtein et al. recently reported the use of urethra-sparing IMRT with daily pretreatment orthogonal imaging for set-up [18]. They compared the clinical outcomes between eight patients with normal IMRT and eight patients with urethra dose-sparing IMRT. They reported that there was no PSA failure in the normal IMRT patients, but 3 of the 8 patients in the urethra dose-sparing IMRT group experienced PSA relapse. The three PSA relapses were at the peripheral zone at the opposite side of the primary site, and not in the dose reduction area around the urethra. Therefore, it cannot be concluded from the Vainshtein et al. study that the urethra dose-sparing is the direct cause of PSA recurrence. In addition, as those authors noted, the intra-fractional position correction was not performed in their treatment; this could be a cause of the high PSA failure rate and may have had no benefit in reducing GU toxicity. The present study suggests that urethra dose-sparing IMRT itself is not a harmful as long as both proper treatment planning procedure and intra-fractional position correction are used.

For AEs, Fonteyne et al. recently reported that probability of developing Grade2, Grade3/4 GI and GU AEs [19], at the point of 24-36month actuarial risk may indicate us the outlook for the incidence of developing long term AEs. However, we must be careful since there is a tendency of increasing GU toxicity with time even after 6–8 years. It should be noted that the median follow up period of this study is still too short to make any firm conclusion of the long term results for both bRFS and AEs.

## Conclusions

IMRT with urethra dose reduction and precise target localization during irradiation using intra-IGRT for intra-fractional guidance achieved very low incidences of acute and late GI and GU toxicity. In addition, the small margin around the CTV and the dose reduction around the urethra did not result in a high PSA failure rate. We have elucidated the clinical benefits of on-line image guidance with the precise correction of the table position during radiotherapy.

## Abbreviations

AE: Adverse event; BED: Biological effective dose; bRFS: Biochemical relapse-free survival; CT: Computed tomography; CTCAE v4.0: Common Toxicity Criteria Adverse Events version 4; CTV: Clinical target volume; GI: Gastrointestinal; GU: Genitourinary; IGRT: Image-guided radiation therapy; IMRT: Intensity-modulated radiation therapy; MRI: Magnetic resonance imaging; NCCN: National Comprehensive Cancer Network; OAR: Organs at risk; PRV: Planning, organ at risk volume; PSA: Prostate-specific antigen; PTV: Planning target volume; ROI: Region of interest; RTRT: Real-time tumor-tracking radiation therapy; TPS: Treatment planning system.

## Competing interests

Dr. Shirato has a patent US 6,307,914 B1 (Oct.23, 2001) “MOVING BODY PURSUIT IRRADIATING DEVICE AND POSITIONING METHOD USING THIS DEVICE” licensed to Hokkaido University, Japan. The other authors declare that they have no competing interests.

## Authors’ contributions

SS conceived of the study. HS. supervised the project and is guarantor of the data. SS, KN, RK, NK, and RO recruited, treated, and followed up the patients. SS, KN and RS analyzed the data. SS. wrote the first draft of the paper. NS, TA, and SM inserted the fiducial markers and supported the patient follow-up. All authors contributed to the drafting and editing of the manuscript and approved the final version.
